# The *in vivo* fate of ^225^Ac daughter nuclides using polymersomes as a model carrier

**DOI:** 10.1038/s41598-019-48298-8

**Published:** 2019-08-12

**Authors:** R. M. de Kruijff, R. Raavé, A. Kip, J. Molkenboer-Kuenen, A. Morgenstern, F. Bruchertseifer, S. Heskamp, A. G. Denkova

**Affiliations:** 1 Radiation Science and Technology, Delft University of Technology, Delft, The Netherlands; 2 Radiology and Nuclear Medicine, Radboud university medical center, Nijmegen, The Netherlands; 3 European Commission, Joint Research Centre, Directorate for Nuclear Safety and Security, Karlsruhe, Germany

**Keywords:** Preclinical research, Nuclear chemistry, Radiotherapy

## Abstract

Increasing attention is given to personalized tumour therapy, where α-emitters can potentially play an important role. Alpha particles are ideal for localized cell killing because of their high linear energy transfer and short ranges. However, upon the emission of an α particle the daughter nuclide experiences a recoil energy large enough to ensure decoupling from any chemical bond. These ‘free’ daughter nuclides are no longer targeted to the tumour and can accumulate in normal tissue. In this paper, we used polymersomes as model carrier to evaluate the retention of recoiling daughters of ^225^Ac *in vivo*, and assessed their suitability as therapeutic agents. Vesicles containing ^225^Ac were injected intravenously in healthy mice, and intratumourally in tumour-bearing mice, and the relocation of free ^213^Bi was assessed in different organs upon the injection [^225^Ac]Ac-polymersomes. The therapeutic effect of ^225^Ac-containing vesicles was studied upon intratumoural injection, where treatment groups experienced no tumour-related deaths over a 115 day period. While polymersomes containing ^225^Ac could be suitable agents for long-term irradiation of tumours without causing significant renal toxicity, there is still a significant re-distribution of daughter nuclides throughout the body, signifying the importance of careful evaluation of the effect of daughter nuclides in targeted alpha therapy.

## Introduction

Personalized medicine is a rapidly growing field in cancer therapy research. The use of tumour-specific therapeutics has proven to significantly increase patient survival and decrease side effects. A number of β-emitting radiotherapeutics are routinely being used for the treatment of e.g. metastasized prostate cancer and neuroendocrine tumours^1,2^. An increasingly popular alternative can be found in the use of α-emitting radionuclides. Only a few α particles passing through the nucleus are sufficient to cause multiple double-strand breaks in the DNA and subsequent cell death. Due to their much higher linear energy transfer (LET), α-emitters are more cytotoxic than β-emitters^3^. Other advantages of the use of α-emitters in tumour therapy include their independence on tumour oxygenation, and their short tissue range which prevents damage to neighbouring healthy cells.

Thus far, promising preclinical and clinical results have been reported for a number of α-emitters, including ^213^Bi (t_1/2_ = 45.6 min) which has successfully been used to treat a different tumours^4,5^. A lot of attention is currently being paid to ^225^Ac (t_1/2_ = 10 days), which provides clear advantages over ^213^Bi including a long half-life allowing more time for accumulation at the tumour site. Furthermore, 4 α particles are emitted in the decay chain of ^225^Ac, resulting in a larger dose to the tumour site per mother nuclide. A number of successes have been realized in clinical trials using ^225^Ac, including treatment of neuroendocrine tumours, prostate cancer, and gliomas^5^. The use of ^225^Ac-PSMA-617 for the treatment of metastatic castration-resistant prostate cancer shows some particularly promising results^6^. Even upon progressive disease during after 2 cycles of ^177^Lu-PSMA-617, impressive treatment response to treatment with ^225^Ac-PSMA-617 were observed^7^. However, one of the potential issues arising when using ^225^Ac for therapy is unwanted toxicity from recoiled daughter radionuclides. Upon the emission of an α particle the radioactive daughter nuclides experience a recoil energy of about 100–200 keV^8^ which is much larger than the energy of any chemical bond and will thus always result in the daughter nuclide breaking free from the targeting agent. Furthermore, the different chemical properties of the daughter nuclide can make re-association with the chelator very unlikely^9^. These ‘free’ daughter nuclides can be a source of dose limiting toxicity^10^. For instance, long-term renal toxicity ﻿has been observed in a study using ^225^Ac labelled anti-rat HER-2/neu monoclonal antibody due to the relocation of recoiling daughter atoms to the kidney^11^. In another study by Kennel *et al*., the potential of killing lung tumours *in vivo* was clearly demonstrated but at the cost of radiotoxic effects associated with released daughter radionuclides which were likely partially responsible for the death of the animals^12^.

Encapsulation of the ^225^Ac mother nuclide in nanocarriers can help retain the daughter atoms at the tumour site and thus limit damage to healthy organs. This approach has been investigated with a number of nanoparticles, including metal-based particles^13^, zeolites^14^ and liposomes^15^. Woodward *et al*. demonstrated that LaPO_4_ based nanoparticles are capable of containing ^225^Ac daughter nuclides. They observed a near complete retention of ^225^Ac in the nanoparticles, but found that about 50% of the daughter nuclides were released *in vitro*. Subsequently, they followed the *in vivo* release of ^213^Bi in the lungs, liver, spleen and kidney. Elevated levels of ^213^Bi were found in the kidneys, indicating some release of this daughter nuclide following nuclear recoil, despite its encapsulation in the nanoparticle^16^. ^225^Ac-containing liposomes have been shown to be very efficient at selectively killing tumour cells *in vitro*^17^. Their efficacy has been tested in 3D tumour models as well as *in vivo*, introducing a diffusion-assisted approach for full tumour coverage. Here, the liposomes degrade upon entering the lower-pH tumour environment, releasing ^225^Ac and its daughter nuclides and hence allowing for a better distribution *in vitro*. This study has shown promising *in vivo* results, with a significant decrease in tumour volume upon the intravenous administration of the liposomes^18^. The distribution of the daughter atoms *in vivo* was not assessed, nor was potential renal toxicity considered, however, very poor ^213^Bi retention in 100 nm liposomes has been reported in an earlier *in vitro* study^19^, suggesting similar problems to occur *in vivo*.

The aim of our study is to assess the retention of the radioactive daughter atom ^213^Bi in a carrier system which has demonstrated good retention of the ^225^Ac mother nuclide, and determine the *in vivo* fate of free ^213^Bi^20^. We have selected polymersomes as delivery vesicles for ^225^Ac-based targeted α therapy^8,20,21^, which have shown to retain the daughter nuclides ^221^Fr and ^213^Bi to a certain extent^20^. This retention can be improved by forming small InPO_4_ nanoparticles around the ^225^Ac encapsulated within the polymersomes^21^. Nanocarriers are known to accumulate in the liver and spleen, whereas free ^213^Bi accumulates in the kidneys, allowing for clearly distinguishable uptake characteristics between the polymersomes themselves and any free daughter nuclides. In this study, we evaluate the retention of ^213^Bi, one of the daughters in the decay chain of ^225^Ac, *in vivo* upon intravenous and intratumoural injection, and looked at the distribution of free ^213^Bi in select tissues, This is especially important to consider when using long-circulating carriers like nanoparticles or antibodies for tumour targeting. We assessed the effect of the polymersome diameter as well as the presence of InPO_4_ nanoparticles encapsulated within the vesicles on the retention and distribution of ^213^Bi. Finally, the effect of [^225^Ac]Ac-polymersomes and [^225^Ac]AcDOTA control on tumour growth, proliferation, apoptosis, and double strand DNA breaks upon intratumoural injection are evaluated in BALB/c nude mice.

## Results and Discussion

It is well known that the recoil energy experienced by the daughter atom is sufficient to break any chemical bond between the daughter atom and a targeting vector. This issue could partially be circumvented through the use of nanocarriers, such as polymersomes, which present a system which is capable of retaining at least part of the daughter nuclides. We have used polymersomes to encapsulate ^225^Ac in the aqueous cavity of the vesicles containing either DTPA^20^ or InPO_4_ nanoparticles^21^. In this study, we compared the recoil retention of the ^213^Bi daughter nuclide *in vivo* of both these polymersome formulations. Throughout this manuscript, ‘free ^213^Bi’ indicates ^213^Bi which is no longer encapsulated in the polymersomes following nuclear recoil.

### Vesicle characterization and loading of radionuclides

**Figure 1:**
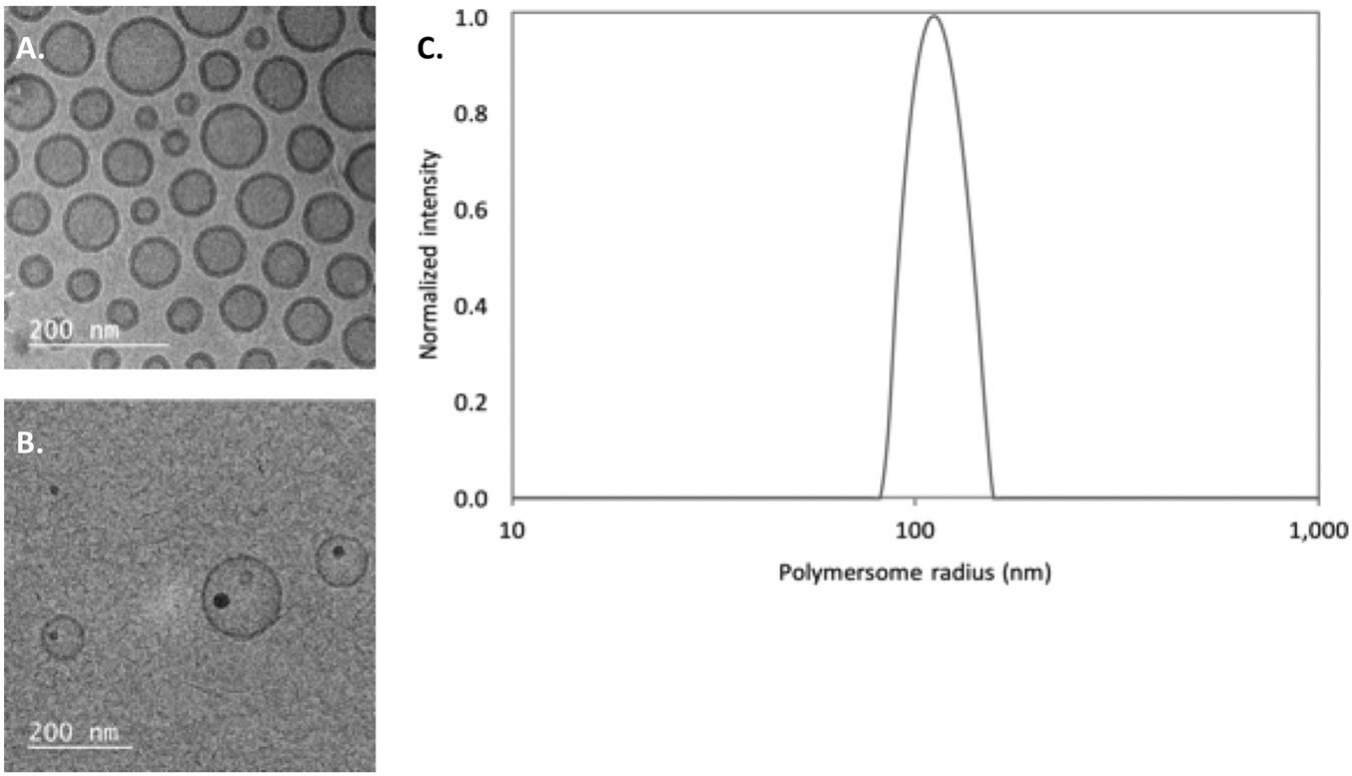
Cryo-TEM images of 100 nm diameter polymersomes containing DTPA (**A**) or InPO_4_ (**B**), and a representative DLS measurement of 100 nm diameter polymersomes (**C**).

Prior to using the polymersomes in the *in vivo* studies, they were fully characterized by both DLS and Cryo-TEM (Fig. 1). In all cases, relatively monodisperse particles were observed, with an average diameter of 97 ± 37 nm as measured by CryoTEM. As mentioned before by Wang *et al*., DLS measurements of the average diameter yield a slight overestimation compared to the CryoTEM images^22^. The InPO_4_ nanoparticles (18 ± 9 nm in diameter) could also be nicely visualized within the polymeric nanocarriers.

Polymersomes were loaded with ^225^Ac according to the well-documented procedures as described earlier by Wang *et al*.^20^ and de Kruijff *et al*.^21^. A loading efficiency of 54 ± 21% has been achieved in the DTPA-containing polymersomes, and 59 ± 6% for co-precipitation in InPO_4_ containing polymersomes. The labelling efficiency of the [^225^Ac]AcDOTA compound used for intratumoural injection was 92.3%.

### Recoil retention ^213^Bi

#### Intravenous injection

We assessed the two different polymersome formulations, ^225^Ac chelated with DTPA or ^225^Ac coprecipitated with an InPO_4_ nanoparticle, on the *in vivo* recoil retention of ^213^Bi upon intravenous injection. While the ^225^Ac mother nuclide is very well retained in both systems (retention of approximately 93%)^20,21^, this is not the case for the daughter radionuclides. The theoretical recoil distance of the daughter radionuclides is about 100 nm in water^8^, which means that the probability of the daughter nuclides to be being retained in polymersomes with an optimal diameter of around 100 nm is limited^23,24^. The use of larger polymersomes will increase the retention of daughter radionuclides, but this is known to reduce the circulation time and subsequent accumulation in tumour tissue^21^. Another factor influencing the retention of recoiled daughter is the location of the decaying radionuclide in the vesicle. For instance, if the mother nuclide ^217^At (second daughter of ^225^Ac) is located in the polymersome bilayer, the chance of the ^213^Bi daughter recoiling out of the vesicle is much higher than when ^217^At would be located in the centre. The loading of ^225^Ac into polymersomes through co-precipitation with InPO_4_ nanoparticles has been shown more successful in retaining daughter nuclides compared with ^225^Ac loaded through chelation with DTPA^21^. When ^225^Ac is co-precipitated with InPO_4_, the recoil distance decreases to about 30 nm, greatly increasing the probability of daughter retention.

**Figure 2:**
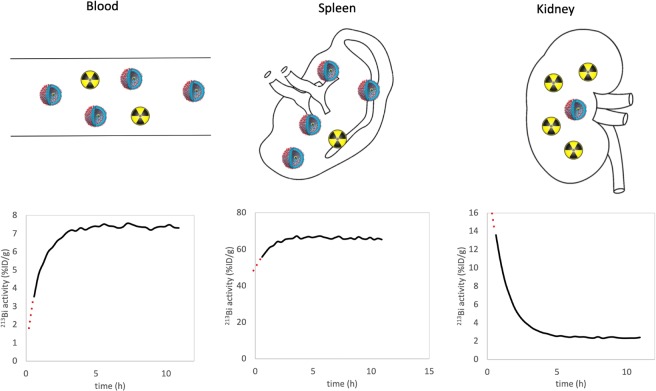
Schematic depictions of the amount of polymersomes () containing ^225^Ac and associated daughter nuclides, and free ^213^Bi () in three organs (blood, spleen and kidney), with underneath per organ the measured % ID/g activity of ^213^Bi as function of the measurement time. Time t = 0 represents the moment of sacrifice, 4 h after the injection of 50 kBq 100 nm [^225^Ac]Ac-polymersomes. The percentage of free ^213^Bi daughters can be obtained by extrapolating back to t = 0, and dividing by the equilibrium activity.

To determine whether these predictions also hold within a more complex *in vivo* system, the distribution of ^213^Bi after administration of [^225^Ac]Ac-polymersomes was determined in mice. With a half-life of a little under an hour, the ingrowth of ^213^Bi was determined through continuous measurement of the *ex-vivo* organ activity in time. These measurements allowed for extrapolation to the time of sacrifice and a direct comparison of the presence of ^213^Bi and ^225^Ac at organ level (see Fig. 2).

Unfortunately, given both the short half-life of ^221^Fr (t_1/2_ = 4.8 min) and the time it took to perform the biodistributions together with the relatively large distance between the animal facility and gamma counter, we were not able to accurately assess the distribution of ^221^Fr. Like most nanocarriers, polymersomes accumulate mainly in the spleen and liver^25^, while free ^213^Bi is known to accumulate mainly in the kidneys (40%), or is excreted via the urine (30%)^26^. To be able to measure the ^213^Bi ingrowth with a certain degree of accuracy, it was important to keep the total number of samples small so that they could be measured frequently. To this end, it was decided to determine the recoil retention of ^213^Bi in three organs of interest; the blood (injection site), spleen (polymersome accumulation) and kidneys (accumulation of free ^213^Bi). Immediately upon sacrifice, the organs of interest were collected and continuously measured until the daughter nuclides were in equilibrium with the mother nuclide ^225^Ac. Back-extrapolation of the obtained data enabled the determination of the amount of ^213^Bi at the moment of sacrificing the animal, as this is the time point at which blood flow stops, stopping subsequent inter-organ redistribution of free daughters. The activity at this time (${A}_{{213}_{Bi}}(t=0)$) therefore represents the activity of ^213^Bi nuclides both from within the polymersome, as well as any redistributed free ^213^Bi. At equilibrium, the ^213^Bi activity (${A}_{{213}_{Bi}}(t=eq)$) comes only from the decay of ^225^Ac in the polymersomes, and thus gives an accurate representation of the biodistribution of the polymersomes. The ratio of these two numbers indicates the distribution of free ^213^Bi, and shows that the redistribution of daughter nuclides has a non-negligible effect on the dose distribution of α therapy to the different organs.

In Table 1, the activity of ^213^Bi at the moment of sacrifice (t = 0) as well as in equilibrium with ^225^Ac is given for selected organs. Furthermore, the ratio of the ^213^Bi at t = 0 to the equilibrium activity of ^213^Bi after in-growth to the mother nuclide activity is given. When this ratio is <1, it means that the organ of interest is releasing ^213^Bi (in this case the blood and spleen), while for a ratio >1, the organ is accumulating free ^213^Bi. The daughter nuclide distribution presented in Table 1 was measured 4 h after the injection of ^225^Ac encapsulated in either DTPA-containing polymersomes, or co-precipitated in InPO_4_ nanoparticles within the polymersomes. In both cases, a significant difference (p < 0.02) between initial ^213^Bi presence and ^213^Bi levels at equilibrium with ^225^Ac was found in the blood. Significantly more (p < 0.05) ^213^Bi was retained in the blood for the InPO_4_ containing vesicles vs the DTPA ones (with ratios of 0.14 ± 0.07 and 0.06 ± 0.03 respectively). Based on the blood values, co-precipitation of ^225^Ac with InPO_4_ nanoparticles in polymersomes constitutes a definite improvement over DTPA containing polymersomes. However, the ratio of the redistributed ^213^Bi at time of sacrifice to the equilibrium ^213^Bi activity found in the spleen and to the kidneys is not significantly different (at a significance level of α = 0.05) when considering the two different polymersome formulations. At first glance this is surprising; a lower amount of free ^213^Bi would be expected to be present in the mice injected with InPO_4_-containing polymersomes based on the blood values, which should have resulted in ratios closer to 1 for both the spleen and the kidneys. However, in the kidneys the accumulation of InPO_4_-containing polymersomes is nearly a factor two lower than for the DTPA-containing polymersomes (2.6 ± 1.1% ID/g vs 4.7 ± 0.8% ID/g respectively), which subsequently increases the ratio of free ^213^Bi to the equilibrium activity of ^213^Bi in polymersomes to similar levels. Free ^213^Bi present in the kidneys at time t = 0 nearly exclusively originates from the release of daughter nuclides from the polymersomes in other organs. The amount of free ^213^Bi in the kidneys at time of sacrifice therefore more accurately represents the amount of recoiled ^213^Bi than the ratio does. There is significantly less free ^213^Bi at t = 0 in the kidneys for the InPO_4_ containing polymersomes (at a significance level of α = 0.05) which agrees with the differences observed in the blood. For the spleen, similar ratios between the DTPA and InPO_4_ containing polymersomes were observed. This is most likely explained by the fact that macrophages within the spleen take up the polymersomes, and the macrophages themselves can than act as a secondary barrier against the escape of the ^213^Bi daughter nuclides from the macrophages. This effect has been described by McDevitt *et al*., who used ^225^Ac-labeled antibodies, and found that upon internalization in the cell, the cell aids in the retention of the daughter nuclides^27^.

**Table 1:** Fraction of free ^213^Bi (recoiled out of the polymersomes and redistributed to other organs) to ^225^Ac in 100 nm polymersomes at 4 h p.i. in the blood, spleen and kidneys, calculated as $Ratio=\,{A}_{{213}_{Bi}}(t=0)/{A}_{{213}_{Bi}}(t=eq)$, with ${A}_{{213}_{Bi}}(t)$ the ^213^Bi activity at time of death (t = 0) or in equilibrium with the mother nuclide ^225^Ac (t = eq).

Organ	Blood		Spleen		Kidney	
	DTPA	InPO_4_	DTPA	InPO_4_	DTPA	InPO_4_
${A}_{{213}_{Bi}}(t=0)$ (% ID/g)	1.1 ± 0.4	1.1 ± 0.4	58 ± 10	44 ± 12	36 ± 5	25 ± 7
${A}_{{213}_{Bi}}(t=eq)$ (% ID/g)	17.9 ± 2.6	8.8 ± 4.5	86 ± 17	69 ± 15	4.7 ± 0.8	2.6 ± 1.1
*Ratio*	0.06 ± 0.03	0.14 ± 0.07	0.67 ± 0.02	0.64 ± 0.06	7.6 ± 0.6	10.5 ± 3.6

^225^Ac was encapsulated in polymersomes containing either the hydrophilic chelate DTPA or InPO_4_ nanoparticles. ﻿The uncertainty corresponds to standard deviations based on five mice per polymersome type.

In this study, we have assessed the retention of ^213^Bi in the polymersomes upon intravenous injection, and looked at the distribution of free ^213^Bi in organs of interest. However, whereas the daughter radionuclides are retained to at least some degree within the polymersomes themselves, they will always be released when chelated to a tumour-targeting antibody due to the recoil effect^28^, with amongst others the different chemical properties of the daughter nuclide making re-association very unlikely. Therefore, it is of great interest to compare the results obtained in our study to antibody-targeting studies assessing the recoil retention *in vivo*. Unfortunately, to date there are only very few studies looking at the free daughter distribution upon decay of ^225^Ac attached to an antibody. McDevitt *et al*. recognized that the retention of the daughter alpha-emitters at the target site is critical to the success of the therapy, and therefore specifically focussed on the use of target cell-internalized ^225^Ac constructs, where internalization by the tumour cell itself resulted in enhanced retention of the daughter nuclides^27^. The daughter nuclides ^221^Fr and ^213^Bi were very well retained at the tumour site at 2 days p.i. (88 ± 9% and 89 ± 2% respectively), although it has to be mentioned that these tumours were counted between 6 and 12 minutes after death and not extrapolated back to time of death, increasing the calculated tumour retention. Kidney values of ^213^Bi show a significant accumulation of free ^213^Bi, at least 3 times higher than at equilibrium. However, the equilibrium values of the ^225^Ac-antibody constructs in the kidneys are much higher compared to the low uptake of [^225^Ac]Ac-polymersomes found in our study, which significantly decreases the ratio of free ^213^Bi to that in equilibrium with ^225^Ac. Furthermore, their values were reported in cpm instead of % ID/g preventing the direct comparison of free ^213^Bi uptake. Another study by Jaggi *et al*. looked in detail at the distribution of ^225^Ac daughter nuclides upon the intravenous injection of ^225^Ac-labeled antibodies^9^. While in most cases they do not specifically assess the ratio of the free daughter nuclide to the equilibrium situation, they do show a kidney-to-femur ratio of ^225^Ac which is much lower than that of ^213^Bi (an estimated ratio of approximately 1 for ^225^Ac vs 20 for ^213^Bi) indicating significant redistribution of ^213^Bi to the kidneys. Song *et al*. used ^225^Ac-labeled anti-rat HER-2/neu monoclonal antibody and found a difference in kidney activity of ^213^Bi at time of sacrifice of 2449.8 Bq/g to 887.0 Bq/g at equilibrium^11^, also concluding that there is significant uptake of free ^213^Bi in the kidneys. Therefore, while all these studies show that there is indeed redistribution of free ^213^Bi, confirming the results obtained in our study, the presented ratios cannot be compared directly.

#### Intratumoural injection

To determine the distribution of free daughter nuclides after accumulation in the tumour, ^225^Ac-containing polymersomes were intratumourally injected and the distribution of ^213^Bi was quantified immediately upon sacrifice. In these experiments, ^225^Ac was encapsulated into polymersomes through chelation with DTPA. Although the polymersomes where ^225^Ac was co-precipitated with InPO_4_ yielded better ^213^Bi retention upon intravenous injection, polymersomes containing DTPA have been studied more extensively^20,22^, including their therapeutic potential *in vitro*^29^, and were thus selected for the intratumoural experiments presented in this paper. Again, free ^213^Bi was expected to be transported by the blood to the kidneys, and hence the organs of interest in this study were the blood, kidneys and tumour. Polymersomes with a diameter of either 100 nm or 200 nm were intratumorally injected to study the effect of polymersome diameter on daughter nuclide retention. These larger vesicles were not used in the intravenous study, as polymersomes should ideally have a diameter below 100 nm^23^ for optimal circulation times.

Table 2 shows the retention of ^213^Bi in the tumour tissue, giving both the amount of ^213^Bi present in tissue at time of sacrifice (t = 0) as well as at equilibrium. The ratios of free ^213^Bi to ^213^Bi in equilibrium are also displayed in this table, where again a ratio is <1 signifies a release of ^213^Bi in the tissue of interest, and a ratio >1 an accumulation of free ^213^Bi. Clearly, the tumour is in all cases the only tissue releasing free ^213^Bi, where the ^213^Bi is subsequently transported by the blood to the kidneys amongst others. However, it has to be kept in mind that the ratios for both the blood and the kidneys appear exceedingly high due to near-zero amount of polymersomes present in these organs at equilibrium. Rather, for these organs the activity of free ^213^Bi at time of sacrifice should be regarded as the main indicator of their free ^213^Bi accumulation. The very low radionuclide activity in the blood shows that ^213^Bi is transported by the blood but not retained there. There was no large difference in free ^213^Bi uptake between the 100 nm and 200 nm vesicles in the blood, although in all cases kidney uptake of free ^213^Bi was slightly lower for the 200 nm polymersomes. This corresponds to the retention of free ^213^Bi in the tumour tissue, where although already quite well retained for 100 nm polymersomes, ^213^Bi is nearly completely retained in the tumour when encapsulated in 200 nm vesicles. However, for both polymersome diameters, no significant increase in tumour retention of ^213^Bi is seen over time, which indicates that the size of the polymersomes still influences the retention of the daughter nuclides suggesting that part of the vesicles has not yet been taken up by the tumour cells. This corresponds to earlier obtained results in *in vitro* tumour spheroids, where the polymersomes were still distributing themselves throughout the spheroid more than 4 days after the addition of fluorescently labelled polymersomes to the cell medium^29^. Looking specifically at potential kidney toxicity, the activity of ^213^Bi at time of sacrifice is in all cases much higher in the tumour than in the kidneys, with tumour: kidney ratios of 9.9 for 100 nm polymersomes, and 29.5 for 200 nm polymersomes at 7 days p.i. Renal toxicity due to free ^225^Ac daughter atoms which have recoiled out of the polymersomes was thus not be expected to be a problem for the therapeutic studies^20^.

**Table 2:** Recoil retention of ^213^Bi in the tumour tissue at 1 and 7 days after intratumoural injection of ^225^Ac encapsulated in polymersomes with a diameter of either 100 nm or 200 nm.

Organ 1 day p.i.	Blood		Tumour		Kidney	
	100 nm	200 nm	100 nm	200 nm	100 nm	200 nm
${A}_{{213}_{Bi}}(t=0)$ (% ID/g)	1.2 ± 0.1	1.5 ± 1.4	122 ± 57	318 ± 111	23.0 ± 5.4	17 ± 7
${A}_{{213}_{Bi}}(t=eq)$ (% ID/g)	0.05 ± 0.01	0.02 ± 0.01	212 ± 100	386 ± 154	0.87 ± 0.09	0.8 ± 0.4
*Ratio*	26 ± 8	58 ± 34	0.58 ± 0.06	0.87 ± 0.04	26 ± 6	23 ± 5
**7 days p.i**.	**100** **nm**	**200** **nm**	**100** **nm**	**200** **nm**	**100** **nm**	**200** **nm**
${A}_{{213}_{Bi}}(t=0)$ (% ID/g)	0.9 ± 0.4	1.8 ± 0.9	146 ± 96	285 ± 75	15 ± 6	9 ± 4
${A}_{{213}_{Bi}}(t=eq)$ (% ID/g)	0.01 ± 0.01	0.3 ± 0.4	269 ± 224	320 ± 116	0.9 ± 0.5	0.8 ± 0.5
*Ratio*	70 ± 40	30 ± 36	0.59 ± 0.12	0.91 ± 0.10	19 ± 10	12 ± 5

The numbers represent the ratio of the ${A}_{{213}_{Bi}}(t)$_,_ the ^213^Bi activity at time t = 0 of sacrifice, to ^225^Ac (t = eq), the ^213^Bi activity in equilibrium with ^225^Ac, calculated as $Ratio={A}_{{213}_{Bi}}(t=0)/{A}_{{213}_{Bi}}(t=eq)$. The uncertainty corresponds to standard deviations based on three mice per polymersome diameter and time point.

A study by Woodward *et al*., examined the recoil retention of ^213^Bi in LaPO_4_ nanoparticles containing ^225^Ac and showed that the release of ^213^Bi from the target organ decreased over the course of 5 days^16^. They attributed this to the potential uptake of their nanoparticle conjugates by the endothelial cells of the lungs, which subsequently trapped any free ^213^Bi within the cells, retarding its diffusion through the tissue. We did not observe a similar pattern in our study; the retention of ^213^Bi in the tumour tissue is stable in time for both polymersome sizes. This difference in behaviour could be due to the different injection methodologies. Woodward *et al*. injected their nanoparticles intravenously allowing them to be taken up in the target tissue in time. Although they did not present any direct measurements of circulation time of the nanoparticles, the activity in the target organs is still increasing up to 48 h p.i., pointing to a long circulation time during which the nanoparticles are not completely taken up in the endothelial cells yet. On the other hand, our polymersomes were injected directly within the tumour tissue and hence did not circulate for an extended period of time, resulting in no significant increase in retention between 1 and 7 days p.i.

### Biodistribution

#### Intravenous injection

**Figure 3:**
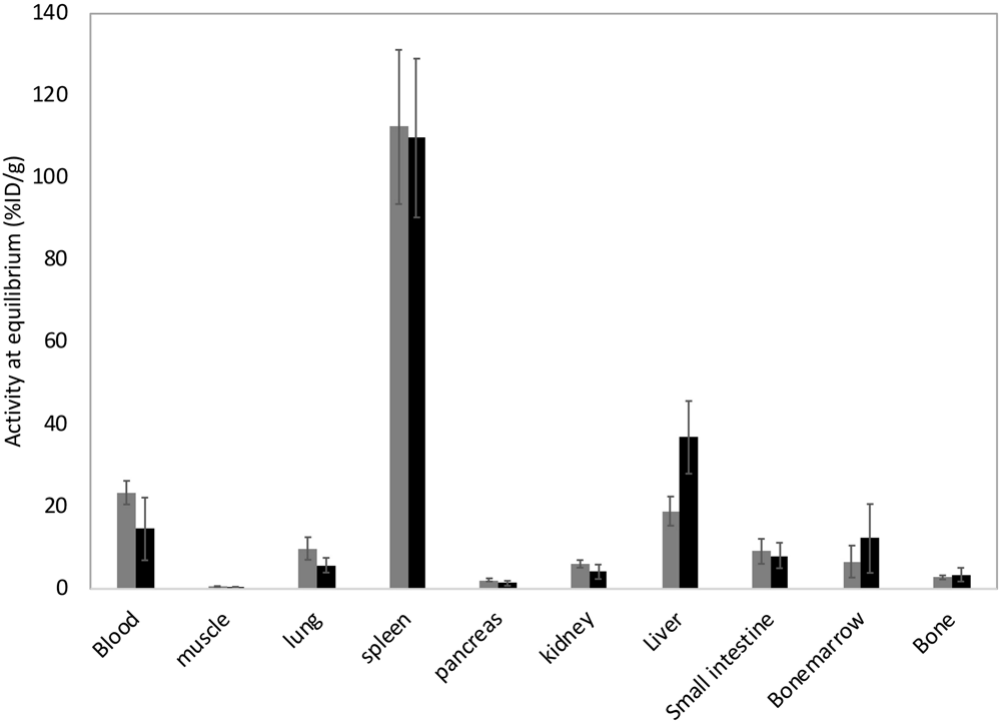
Biodistribution data of ^225^Ac-containing 100 nm diameter polymersomes intravenously injected in healthy female Balb/c nude mice. ^225^Ac was either bound to DTPA in the polymersome (grey), or precipitated with InPO_4_ in polymersomes (black). The ^221^Fr activity at equilibrium is displayed here, and taken as a direct representation of the ^225^Ac activity. Bars represent mean of 5 mice per treatment group with associated standard deviation.

While ^221^Fr emits gamma particles with energies suitable for SPECT imaging^16^, the activity used in this experiment is too low for proper imaging which is why we performed a biodistribution study. The distribution of ^225^Ac complexed to DTPA in polymersomes was compared to that of ^225^Ac co-precipitated with InPO_4_ nanoparticles in polymersomes upon intravenous injection. Biodistribution data of the [^225^Ac]Ac-polymersomes at 4 h p.i. can be seen in Fig. 3, for both ^225^Ac coupled to DTPA or encapsulated in InPO_4_ nanoparticles. Because the radionuclides are encapsulated within the aqueous core of the polymersomes, the outer surface fo the polymersomes remains the same, logically resulting in the similar organ uptake. The only organ displaying a significant difference in uptake between the two types of vesicles is the liver. At the moment, we have no good explanation for this difference in liver uptake. The two types of polymersomes (containing either InPO_4_ or DTPA in the aqueous cavity) were composed of the same batch of block copolymers, prepared according to the same method, and injected in mice which had been randomly distributed between the two groups. Since the outer surface of the polymersomes was not changed, a difference in uptake was not expected, and further research would be required to fully understand this difference in uptake. The circulation time of these polymersomes in tumour bearing mice is known to be considerably shorter than in healthy mice, with circulation half-lives of 5 min and 117 min respectively^30^. The exceedingly short circulation half-life in tumour bearing mice prevents sufficient polymersome accumulation at the tumour site upon the intravenous injection of ^111^In-containing polymersomes (0.44 ± 0.39% ID/g). We therefore decided to assess therapeutic efficacy of [^225^Ac]Ac-polymersomes through intratumoural injection.

#### Intratumoural injection

**Figure 4:**
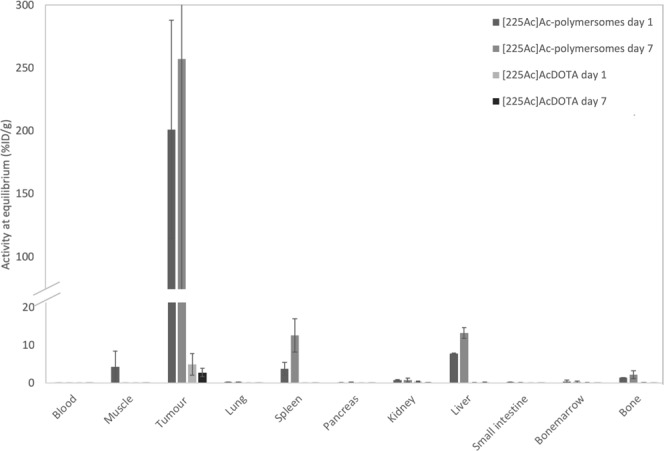
Biodistribution of BALB/c mice bearing an MDA-MB-231 tumour intratumourally injected with 50 kBq ^225^Ac either encapsulated in 100 nm diameter polymersomes, or bound to DOTA, at 1 and 7 days p.i. based on 3 mice per group. The ^221^Fr activity at equilibrium with its parent nuclide ^225^Ac is displayed here, and taken as a direct representation of the ^225^Ac activity. Bars represent mean with associated standard deviation.

For the biodistribution and therapeutic study of intratumorally administered [^225^Ac]Ac-polymersomes, vesicles with a diameter of 100 nm were used. Here, the biodistribution of both intratumourally injected ^225^Ac complexed to DTPA in polymersomes as well as [^225^Ac]AcDOTA was assessed at 1 and 7 days p.i. The reason for the use of [^225^Ac]AcDOTA instead of ^225^Ac-DTPA as a control for the polymersomes is its increased kinetic stability^31^. [^225^Ac]AcDTPA has been shown to display substantial toxicity due to ^225^Ac leakage, whereas [^225^Ac]AcDOTA constitutes an substantial improvement^31^. While this lower stability of the [^225^Ac]AcDTPA complex has been shown not to result in enhanced ^225^Ac leakage from the polymersomes^20^, the more stable [^225^Ac]AcDOTA complex was chosen for the *in vivo* studies to minimize toxicity due to unbound ^225^Ac. In Fig. 4, the distribution of both [^225^Ac]Ac-polymersomes and [^225^Ac]AcDOTA in selected organs is displayed. When viewing these biodistribution results, it is important to keep in mind that the injected activity as well as volume were very small, due to which the activity measured in various organs was just slightly above background. As expected based on earlier results by Wang *et al*.^25^, the polymersomes are very well retained in the tumour tissue, while the [^225^Ac]AcDOTA compound is rapidly cleared. The tumour retention of the polymersomes was found to be 244 ± 74% ID/g and 289 ± 130% ID/g at 1 and 7 days p.i. respectively, whereas less than 10% ID/g and 5% ID/g of the [^225^Ac]AcDOTA was retained at the tumour site at both time-points respectively (Fig. 4).

Uptake in most other organs is minimal for both compounds. In the [^225^Ac]AcDOTA study, nearly no activity is found in any other organ, suggesting that the majority of the compound was excreted already at time of sacrifice. The only organ exhibiting some uptake of [^225^Ac]AcDOTA are the kidneys at 1 day p.i., indicating that renal excretion is indeed the main pathway for [^225^Ac]AcDOTA clearance. At 7 days p.i., renal uptake is present only in insignificant amounts. The [^225^Ac]Ac-polymersomes are much better retained by the tumour tissue than [^225^Ac]AcDOTA. Organs other than the tumour exhibiting uptake of [^225^Ac]Ac-polymersomes are the liver and spleen, and to a lesser extend also the bone and muscle. The main cause for healthy tissue uptake can likely be found in release of the polymersomes themselves. Polymersomes which have diffused out of the tumour tissue will either be filtered out by Kupffer cells, which are responsible for the phagocytic activity of the liver, or by macrophages in the spleen which corresponds well with the uptake observed in Fig. 4 ^32^. The ^225^Ac presence in the healthy organs could also, in part, be caused by ^225^Ac which was released from the polymersomes. Both free ^225^Ac as well as chelated ^225^Ac have been shown to accumulate in the bones as well as in the liver^31,33,34^, and is temporarily also retained in the kidneys^33,35^. Davis *et al*. have shown that this kidney uptake is very time dependent and much smaller than the liver uptake, so even though kidney uptake in our study is minimal, there might be some free ^225^Ac responsible for the organ uptake. However, in earlier work, the ^225^Ac released from the polymersomes was found to be only ﻿2% of the total amount of encapsulated ^225^Ac over a period of 8 days, making the release of ^225^Ac from the polymersomes *in vivo* unlikely^20^. These biodistribution results indicate that renal toxicity from ^225^Ac-polymersome accumulation is unlikely to play a major role in inducing dose-limiting toxicity, though any health issues related to the kidneys and bone marrow should be carefully studied.

### Tumour growth and overall survival upon intratumoural injection

**Figure 5:**
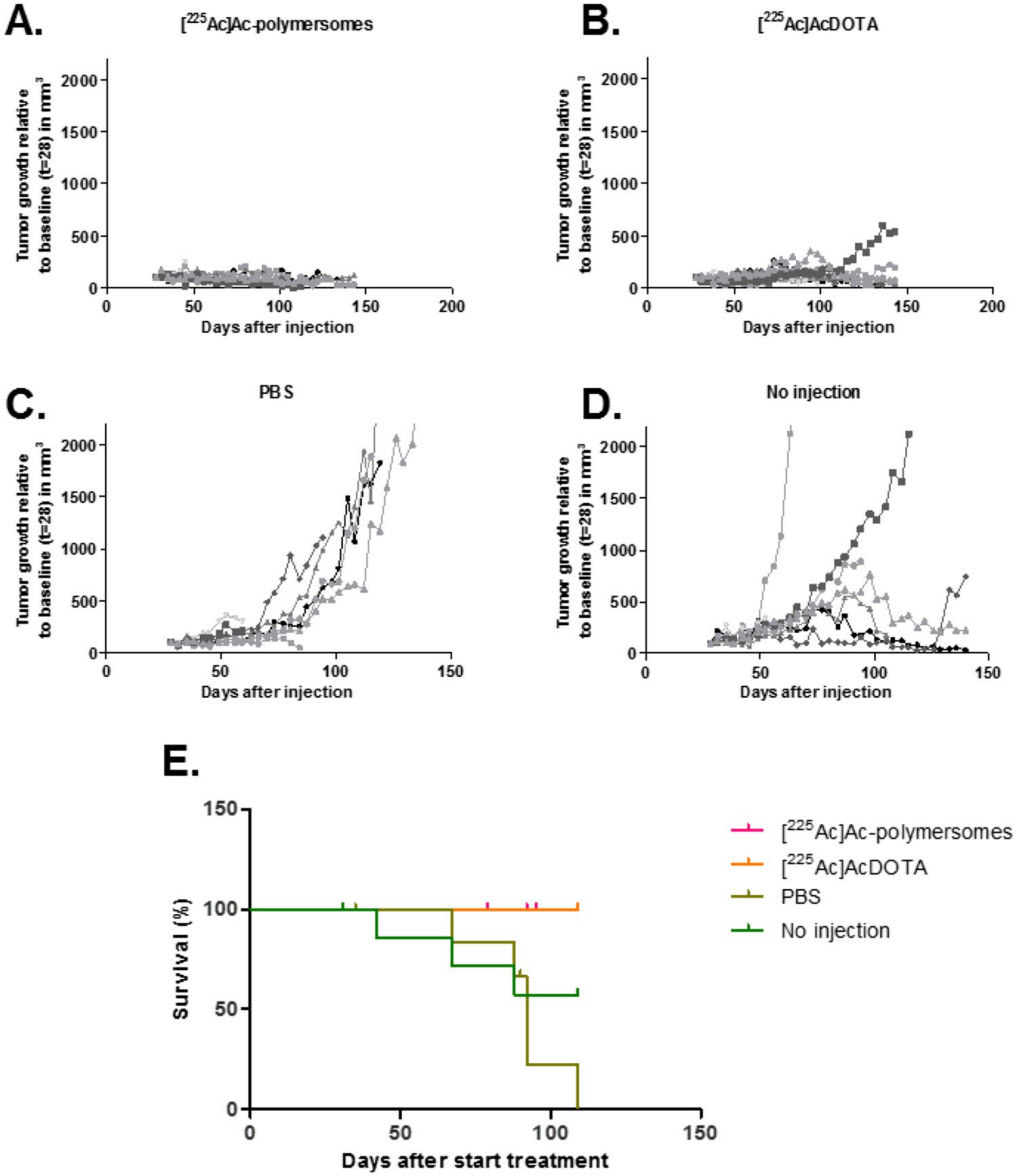
Growth (relative from baseline at day 28 after tumour inoculation) of subcutaneous MDA-MB-231 tumours in female BALB/c mice that received a single intratumoural injection (20 μl) of 50 kBq [^225^Ac]Ac-polymersomes (**A**), 50 kBq [^225^Ac]AcDOTA (**B**), PBS (**C**), or no injection (**D**). The compounds were injected at 25 d after the cell inoculation. Each line reflects an individual mouse (n = 8/group). (**E**) Kaplan-Meier curve of time to tumour related death (*i*.*e*. tumour size > 2000 mm^3^).

The tumour model chosen, MDA-MB-231, is a well-vascularized tumour model. Upon injection of the compounds (the polymersomes as well as the DOTA and PBS), some blood was observed to leak out of the tumours. In Fig. 5, the tumour growth of the 8 mice per group bearing subcutaneous MDA-MB-231 tumours is presented. At 28 days after inoculation the tumours were injected with the various compounds. In 7/8 tumours injected with [^225^Ac]Ac-polymersomes a complete inhibition of tumour growth was observed (Fig. 5A). Two out of eight tumours doubled in size (at day 17 after therapy administration), but decreased in size again over the course of the experiment. A similar tumour growth inhibition pattern was found in mice intratumourally injected with [^225^Ac]AcDOTA; 6/8 tumours with inhibition of tumour growth (Fig. 5B). Five out of eight tumours doubled in size during the experiment (mean time after treatment administration was 67 ± 31 days), but four of these tumours decreased again during the study. Tumours in the two control groups (no intratumoural injection and tumours injected with PBS) showed a pronounced growth starting around 50 days after tumour inoculation (Fig. 5C,D). Seven out of eight tumours were doubled in size at an average of 35 ± 10 days after PBS administration. Six out of eight tumours of the non-injected mice did not show tumour growth inhibition at the end of the study. The average tumour doubling time was 18 ± 9 days after treatment administration, but two of these tumours decreased in size over the course of the experiment. The PBS injection is not expected to have any influence on tumour growth, though the injection itself could have damaged the tumour tissue resulting in regression of the tumour. Surprisingly, tumour regression was also observed in some of the animals which had not received any treatment at all (Fig. 5D), showing that some of these tumours may spontaneously regress. Despite the fact that some tumours from the control groups also showed regression, we could still demonstrate that [^225^Ac]Ac-polymersomes significantly inhibited tumour growth as compared to the control groups. When time to tumour related decease is displayed in a Kaplan-Meier survival curve (Fig. 5E), the therapeutic efficacy of [^225^Ac]Ac-polymersomes and [^225^Ac]AcDOTA administration is evident as demonstrated by significantly (p < 0.05) improved survival as compared with the control groups.

The biodistribution study (Fig. 4) showed that [^225^Ac]Ac-polymersomes are retained very well at the tumour site, whereas [^225^Ac]AcDOTA is cleared rapidly. The tumour dose induced by the 50 kBq [^225^Ac]Ac-polymersomes is roughly equivalent to the dose given by 5 kBq ^225^Ac in polymersomes to a 400 µm tumour spheroid as studied earlier^29^ taking into account the polymersome uptake in the spheroids of only 0.10–0.15%. There, a significant reduction in tumour size was observed at 3–6 days after addition of the vesicles. In the current *in vivo* study, no significant increase in tumour size was observed after the injection of the polymersomes. The excellent response in the group injected with 50 kBq [^225^Ac]AcDOTA, which, similarly to the [^225^Ac]Ac-polymersome group, experienced no tumour related death over the treatment period, was somewhat unexpected. The biodistribution studies at 1 day p.i. show that at this point nearly all [^225^Ac]AcDOTA has been cleared from the tumour site (Fig. 4). However, in previous *in vitro* studies, [^225^Ac]Ac-polymersomes with an activity of only 0.1 kBq, already showed a decrease in tumour growth, indicating that very low amounts of activity can already influence tumour growth^29^. The dose received within the first few hours after injection of [225Ac]AcDOTA was thus sufficient to delay tumour growth, illustrating the effectiveness of α radionuclide therapy.

### Immunohistochemistry

**Figure 6:**
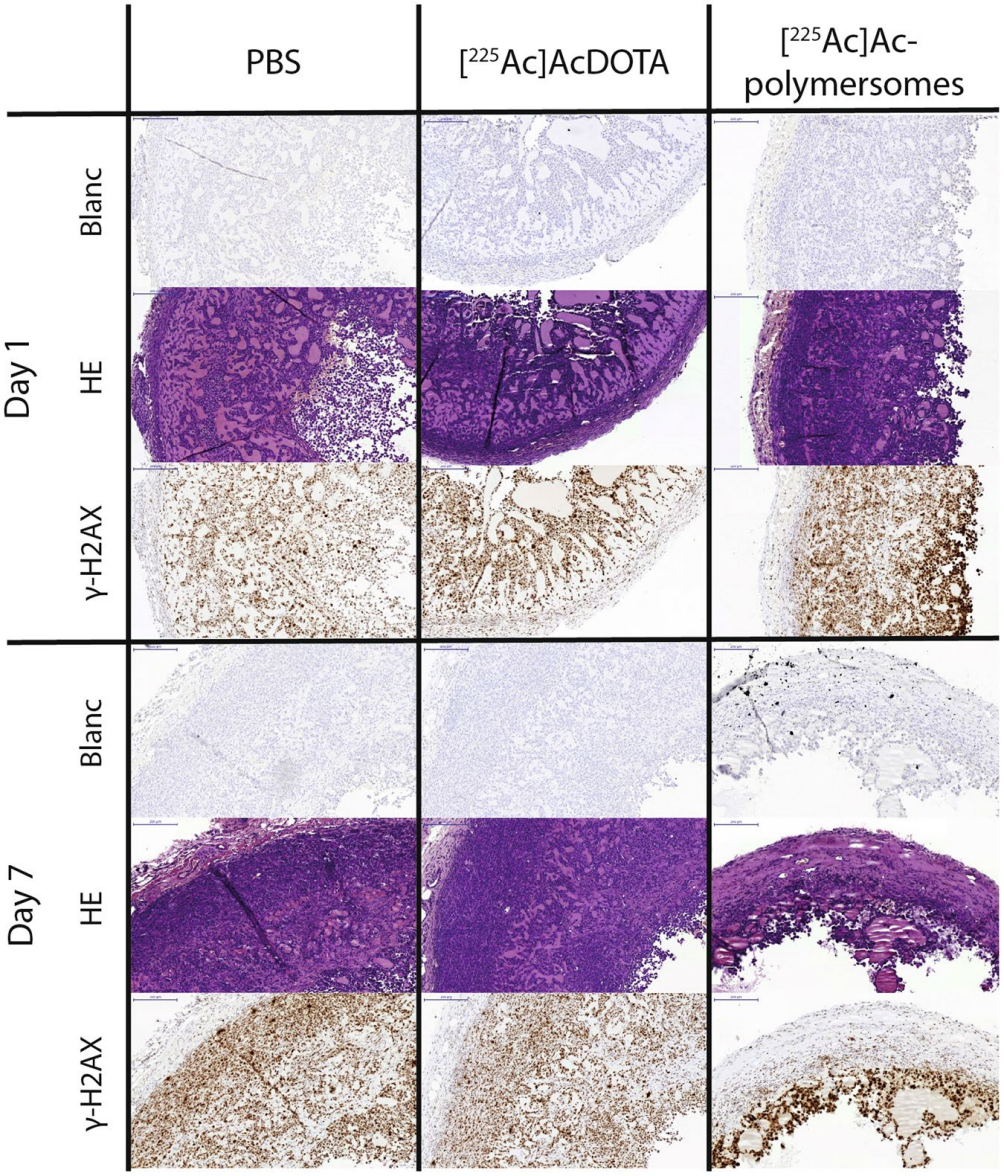
Tumour sections of subcutaneous MDA-MB-231 tumours injected intratumourally with either PBS, [^225^Ac]AcDOTA or [^225^Ac]Ac-polymersomes at 1 and 7 days p.i., where the sections were stained with either HE or γ-H2AX.

To determine the effect of the intratumoural injection of the [^225^Ac]Ac-polymersomes, [^225^Ac]AcDOTA, as well as PBS on the tumour tissue, tissue sections were stained with HE and γ-H2AX (Fig. 6). The HE histological stain was used to demonstrate different tissue structures. While hematoxylin stains nuclei blue, eosin stains the cytoplasm as well as tissue fibers and matrigel^36^. Representative tumour sections of each of the treated groups are presented in Fig. 6. To detect the presence of double-stranded breaks (DSBs) in the DNA, γ-H2AX staining was used. γ-H2AX foci in the tumours gives an indication of the distribution and effectiveness of the α therapy, as the high LET of α radiation causes double strand breaks in DNA^37^. The tumours visualised in Fig. 6 show a similar uniform distribution of γ-H2AX foci in both the PBS and [^225^Ac]AcDOTA groups. The images show that the [^225^Ac]Ac-polymersomes treatment groups demonstrate a larger degree of γ-H2AX foci than the control groups, with an increasing foci gradient towards the centre of the tumour especially at day 1. As the polymersomes were injected in the centre of the tumour, the largest amount of radiation damage logically occurs in the vicinity of the centre. At 7 days p.i. the vesicles have not yet uniformly distributed themselves throughout the tumour, which is in line with the distribution rate as observed in the 3D tumour spheroids^29^, and is expected to improve at later time-points. However, despite the observed therapeutic efficacy in the [^225^Ac]AcDOTA group, the increase in DSBs was less pronounced. This could be due to the fast clearance of the [^225^Ac]AcDOTA complex from the tumour, and a subsequent decline in γ-H2AX signal to detect the DSBs. While the maximum number of γ-H2AX foci is reached 30 min after irradiation, the signal subsequently continuously decreases, reaching half their maximum already 2.5 h p.i.^38,39^. Hence, all damage to the tumour caused by [^225^Ac]AcDOTA will likely had happened within the first few hours p.i., whereas the treatment with [^225^Ac]Ac-polymersomes clearly irradiated the tumour over an extended period of time. Renal toxicity was not observed in any of the treatment groups, confirming the hypothesis that the amount of free ^213^Bi accumulated in the kidneys was not sufficient to cause long-term toxicity issues.

## Conclusion

The main objective of this paper was to study the distribution of free ^213^Bi *in vivo* after either intravenous or intratumoural injection of ^225^Ac encapsulated in polymersomes. It is well known that the recoil energy of the daughter nuclide upon α decay is sufficient to break any chemical bond, leading sometimes to renal toxicity caused by accumulation of ^213^Bi when using conventional antibody or peptide targeting approaches. Polymersomes have been shown to retain ^225^Ac daughter radionuclides to some extent^21^, and in this study we specifically studied the ^213^Bi retention and free ^213^Bi distribution in different tissues of mice. Two types of polymersomes were examined upon intravenous injection, where ^225^Ac was either encapsulated through conjugation with DTPA, or by coprecipitation with InPO_4_ nanoparticles contained within polymersomes. The benefit of the shorter recoil distance caused by the presence of the nanoparticle was shown, as more than twice the amount of ^213^Bi was retained in the case of the polymersomes containing nanoparticles. Upon intratumoural administration, ^213^Bi was retained in the tumour tissue to a large extend, with tumour: kidney ratios of ^213^Bi of 9.9 and 29.5 for 100 nm and 200 nm diameter polymersomes respectively. The therapeutic potential of intratumorally injected [^225^Ac]Ac-polymersomes was also studied, and although biodistribution studies showed very favourable retention of the polymersomes in the tumour tissue, whereas ^225^Ac coupled to DOTA was rapidly excreted, both formulations inhibited tumour growth. No tumour-related deaths were observed in either treatment group, and immunohistochemical analysis of the treatment groups at 1 d and 7 d p.i. showed an increase in γ-H2AX foci in the [^225^Ac]Ac-polymersomes group, indicating a larger degree of double-stranded breaks.

Concluding, while [^225^Ac]Ac-polymersomes and its daughters are retained at least partly the tumour site upon intratumoural administration showing their effectiveness in α therapy, their circulation time and tumour uptake upon intravenous administration needs further optimization before they can be used to target metastasized tumours. Furthermore, despite the much better retention of daughter nuclides in polymersomes compared to targeting molecules, ^213^Bi is still released and accumulates in the kidneys upon intravenous administration. This demonstrates how essential it is for any studies using ^225^Ac as α therapeutic to take daughter nuclide distribution into account.

## Methods

### Chemicals

The block copolymer PBd-PEO with M_w_ of 1900–900 g/mol was purchased from Polymer Source (Quebec, Canada). The ^225^Ac was obtained from the Directorate for Nuclear Safety and Security (Karlsruhe, Germany). The PD10 size exclusion columns were obtained from GE Healthcare (Hoevelaken, the Netherlands). Instant Thin-Layer Chromatography (iTLC) strips were purchased from Varian (USA). For the immunohistochemical analysis, rabbit-anti-H2AX (Cell Signaling, art.nr. 9718), goat-anti-rabbit (Vector, art.nr. BA-1000), avidin-biotin (Vectastain, art.nr. PK-6100) and Bright DAB (Immunlogic, art.nr. BSO4-500) were used. All other chemicals were purchased at Sigma Aldrich.

### Polymersome preparation and loading of ^225^Ac

Polymersomes containing either DTPA as hydrophilic chelator, or KH_2_PO_4_ as precipitating agent were prepared for the *in vivo* studies, according to earlier published procedures^21^. The vesicles used in the intravenous injection studies were prepared using the direct dissolution method^22^, where 10 mg/mL block copolymer was added to a 1 mM DTPA PBS solution at pH 7.4, or a 0.5 M KH_2_PO_4_ PBS solution at pH 3. The solution was stirred at 300 rpm for a week, upon which the polymersomes were extruded to a diameter of 100 nm by passing them several times through polycarbonate filters with cut-off membrane of 800, 400, 200 and 100 nm. For the intratumoural experiments, the DTPA containing vesicles were prepared according to the solvent displacement method. In short, 1 mM DTPA in 1 mL PBS buffer solution (pH 7.4) was slowly added to 1 mL acetone containing 20 mg/mL block copolymer under continuous stirring. After evaporation of the acetone, 1 mL PBS was added to bring the final concentration to 10 mg/mL block copolymer. In all cases, before loading the polymersomes with the radionuclide ^225^Ac, the remaining unencapsulated DTPA or KH_2_PO_4_ was removed by passing it through a Sephadex G 25 size extrusion column (L × D = 30 × 1 cm). ^225^Ac dissolved in pH 2 HCl together with 200 μL 10 mM HEPES buffer was added to a vial containing 0.1 mg A23187 dissolved in 100 μL CHCl_3_. Upon the evaporation of the CHCl_3_, 800 μL polymersome solution was added and incubated for 1 hour. A PD10 column was used for purification, and the 7^th^ 0.5 ml fraction was collected for further *in vivo* experiments at an ^225^Ac concentration of either 250 kBq/mL for the intravenous experiments, or 2 MBq/mL for the intratumoural experiments. Loading efficiencies were calculated as the amount of activity eluted from the PD10 column in the polymersome-containing fractions divided by the total activity in the sample. The daughter nuclide ^221^Fr (218 keV) was counted with the Wizard gamma counter (PerkinElmer) when equilibrium was reached and decay-corrected as a representative measurement of ^225^Ac in the sample. Samples were counted for 1 minute each, with as counting window 170–270 keV for ^221^Fr and 380–520 keV for ^213^Bi.

### DLS and Cryo-TEM measurements

Before being used for the *in vivo* studies, the vesicles were characterized by both dynamic light scattering (DLS) and cryogenic transmission electron microscopy (Cryo-TEM). The DLS consisted of a JDS Uniphase 633 nm 35 mW laser, a fibre detector, an ALV sp 125 s/w 93 goniometer and a Perkin Elmer photon counter, with an ALV-5000/epp correlator and software. A 0.01 mg/mL polymersome solution was placed in a toluene filled, temperature regulated bath (20 °C), where the intensity autocorrelation function was determined at 90°, and the data was fitted using the Contin method. The hydrodynamic radius was obtained using Einstein-Stokes equation.

Cryo-TEM images were obtained by depositing 4 μL of the 10 mg/mL polymersome solution on a holey carbon film (Quantifoil 1.2/1.3, Cu 200 mesh grids) supported on a TEM grid. The drop was blotted for four seconds with filter paper in order to obtain a thin layer on the grid, and subsequently vitrified by rapidly immersing in liquid ethane (Leica EM GP version 16222032). The sample was inserted into a cryo-holder (Gatan model 626) and then transferred to a Jeol JEM 1400 TEM. Images were obtained at an acceleration voltage of 120 keV. For the statistical analysis of the polymersome and nanoparticle diameters, about 30–50 images were made of each of the polymersome samples. The diameter of polymersomes and nanoparticles within those images were measured with ImageJ^40^.

### [^225^Ac]AcDOTA radiolabelling

For the radiolabelling of [^225^Ac]AcDOTA, 1.4 MBq ^225^Ac was added to 8 · 10^−9^ mol DOTA in 40 μL 0.1 M TRIS buffer at pH 9.0. After an incubation time of 1 h at 37 °C the labelling efficiency was determined with iTLC. A 2 µL droplet of the radiolabelled sample was placed on a 7 cm ITLC strip, with 0.1 M NaOH at pH 12 as mobile phase. The ITLC strip was subsequently imaged with the Phosphor-imager to determine the labelling efficiency, which was calculated as the fraction of the total activity which travelled with the mobile phase divided by the total activity on the sample. The sample was used without further purification, and diluted in PBS to an ^225^Ac concentration of 250 kBq/mL for intravenous, and 2 MBq/mL for intratumoural studies.

### Cell culture

The human breast cancer cell line MDA-MB-231 was cultured in RPMI-1640 (GIBCO, ThermoFisher Scientific, Waltham, MA, USA) media supplemented with 2 mM glutamine (GIBCO) and 10% fetal calf serum (Sigma-Aldrich Chemie BV), at 37 °C in a humidified atmosphere with 5% CO_2_. Cells were dissociated when 80–90% confluency was reached using 0.05% trypsin (w/v) in 0.53 mM EDTA (Life Technologies) and maintained as proliferating cultures. Mycoplasma contamination was evaluated every four months using a MycoAlert™ mycoplasma detection kit (Lonza, Basel, Switzerland). After thawing, cells remained in culture for a maximum of six months.

### Animal studies

All animal studies were approved by the Dutch central committee on animal research and the local ethical committee on animal research of the Radboud University under protocol 2015-0071, and performed according to the institutional guidelines. 6–8 weeks old female BALB/cAnNRj-Foxn1^nu^/Foxn1^nu^ mice (Janvier Labs, France) were randomly tattooed for identification upon arrival. The mice were acclimatized for ≥4 days before any experimental procedure, and had unlimited access to food and water. Cages were replaced by clean cages every week, and the animals were housed with 5–6 mice per cage in a controlled environment (12 h dark/light cycle, 22 ± 1 °C, 55 ± 10% humidity). In all biodistribution studies, mice were sacrificed through CO_2_ asphyxiation and the organs of interest were collected, weighed, and the activity of two of the daughter nuclides of ^225^Ac, namely ^221^Fr (218 keV) and ^213^Bi (440 keV), were counted with the Wizard gamma counter (PerkinElmer) approximately 20 hours after sacrifice (when secular equilibrium was reached). The activity concentration for ^225^Ac was estimated from the daughter activity of ^221^Fr counted after equilibrium.

### Daughter nuclide retention upon intravenous injection

Retention of ^213^Bi in the polymersomes was determined *in vivo* through the intravenous injection of 200 μL of the 3 mg/mL polymersome solution containing 50 kBq ^225^Ac in the tail vain of five non-tumour bearing mice per group. Four hours post injection, the mice were sacrificed through CO_2_ asphyxiation. Organs of interest for daughter retention (blood, spleen and kidneys) were rapidly collected and the ^213^Bi activity was measured continuously on the Wizard gamma counter (PerkinElmer) for approximately 18 hours (Fig. 1). ^213^Bi activity at the time of sacrifice was determined by fitting the data with equation 1 and extrapolating back to t = 0^20^:

1 $${A}_{2}(t)={A}_{1}(1-{e}^{-{\lambda }_{2}t})+{A}_{2}(0){e}^{-{\lambda }_{2}t}$$

where *A*_*1*_ the activity of ^225^Ac, and *A*_*2*_*(t)* is the ^213^Bi activity at time *t* with decay constant *λ*_*2*_. Figure 1 depicts the method of extrapolating the ^213^Bi activity in each of the three organs of interest at time of sacrifice. Paired two-sided Student’s t-tests were used to calculate the significance of the difference in organ uptake.

### Intratumoural injection

All mice used in the intratumoural experiments were subcutaneously inoculated with 5 · 10^6^ MDA-MB-231 cells 1:1 in Matrigel. The tumour length, width and height were measured by calliper and the tumour volume was calculated using ${\rm{V}}=\frac{4}{3}\pi (\frac{W}{2})(\frac{L}{2})(\frac{H}{2})$, with V the volume of the tumour, W the width, L the length and H the height of the tumour. Tumour size was used to block-randomized the mice over the different treatment groups. When the average tumour volume was approximately 100 mm^3^, the mice were intratumourally injected with either 25 μL, 50 kBq [^225^Ac]Ac-polymersomes, or 50 kBq [^225^Ac]AcDOTA, and one of the control groups with 25 μL PBS, while the other group was left untreated.

#### Biodistribution and recoil retention

The *ex vivo* biodistribution and ^213^Bi recoil retention were analysed at 1 and 7 days p.i. for the mice having been injected with [^225^Ac]Ac-polymersomes, or [^225^Ac]AcDOTA. The mice of the PBS and untreated groups were sacrificed for immunohistochemical analysis of the tumour and kidneys. Tumours and kidneys of all animals (3 per treatment group) were fixated in 4% paraformaldehyde in PBS overnight and embedded in paraffin to analyse double strand DNA breaks immunohistochemically. Retention of the daughter nuclide ^213^Bi in the tumour, and accumulation in the kidneys, was assessed according to the procedure in the intravenous experiment, where ^213^Bi activity at the time of sacrifice was determined by extrapolating the in-growth back to time t = 0.

#### Therapeutic evaluation

All groups consisted of 8 mice each, of which the tumour growth and overall survival was monitored up to 115 days after the start of treatment. Tumour volume was measured twice a week. Mice were taken out of the experiment when they reached one of the humane endpoints (tumour size > 2 cm^3^, ulceration or invasive tumour growth, other signs of clinical discomfort e.g. 15% weight loss in two days, dehydration). Technicians assessing the humane endpoints were blinded for the treatment groups.

### Immunohistochemistry

Tumours harvested at 1 and 7 days after the intratumoural injection were embedded in paraffin. Immunohistochemistry was performed using 4 µm thick tumour sections. For the haematoxylin and eosin (HE) staining, paraffin was removed by incubation with xylene, and subsequently sections were incubated with haematoxylin for 20 minutes and with eosin for 5 minutes, followed by dehydration and mounting in Permount.

For the staining for γ-H2AX, first antigen unmasking was performed on the deparaffinated tumour sections by treating the slides with 10 mM citrate buffer (pH 6.0) for 10 minutes at 98 °C. After washing the slides with distilled water and PBS, aspecific binding was blocked by incubating the sections with 50–150 µL 5% normal goat serum (NGS) in PBS for 30 minutes at RT. Subsequently, the tumour sections were incubated overnight with 50–150 µL rabbit-anti-H2AX diluted 1000 times in a PBS solution containing 1% BSA (bovine serum albumin) and 5% NGS at 4 °C. Subsequently, slides were washed 3x with PBS and endogenous peroxidase activity was blocked by a 10-minute incubation in 0.3% H_2_O_2_ in methanol at RT. After another two PBS washes, the sections were incubated for 30 minutes at RT with 200 times diluted biotinylated goat-anti-rabbit. Finally, an avidin-biotin complex was applied for 30 minutes at RT. To develop the tumour sections, they were incubated with 50–150 µL Bright DAB for 8 minutes at RT. Nuclei were counterstained with 3x diluted hematoxylin in PBS for 5 seconds. Finally, the sections were dehydrated with consecutively 50%, 70%, and twice 100% ethanol, twice with Xylene, after which they were mounted with Permount, dried and imaged.

### Statistics

Time-to event data collected during the intratumoural efficacy study were analysed using Graphpad Prism (version 5.03, Prism). Statistical significance between groups was analysed by multiple comparisons of the survival curves. Statistical significance was set at p < 0.05 calculated by the Log-rank Mantel-Cox test and corrected for multiple comparisons using a manually calculated Bonferroni-corrected threshold with 6 comparisons and p = 0.05.

## Data Availability

The datasets generated during and/or analyzed during the current study are available from the corresponding author on reasonable request.
